# The development and application of bioinformatics core competencies to improve bioinformatics training and education

**DOI:** 10.1371/journal.pcbi.1005772

**Published:** 2018-02-01

**Authors:** Nicola Mulder, Russell Schwartz, Michelle D. Brazas, Cath Brooksbank, Bruno Gaeta, Sarah L. Morgan, Mark A. Pauley, Anne Rosenwald, Gabriella Rustici, Michael Sierk, Tandy Warnow, Lonnie Welch

**Affiliations:** 1 Computational Biology Division, Department of Integrative Biomedical Sciences, Institute of Infectious Disease and Molecular Medicine, University of Cape Town, Cape Town, South Africa; 2 Department of Biological Sciences and Computational Biology Department, Carnegie Mellon University, Pittsburgh, Pennsylvania, United States of America; 3 Ontario Institute for Cancer Research, Toronto, Canada; 4 EMBL-EBI, Wellcome Genome Campus, Hinxton, Cambridge, United Kingdom; 5 School of Computer Science and Engineering, University of New South Wales, Sydney, Australia; 6 School of Interdisciplinary Informatics, University of Nebraska at Omaha, Omaha, Nebraska, United States of America; 7 Department of Biology, Georgetown University, Washington, DC, United States of America; 8 Department of Genetics, University of Cambridge, Cambridge, United Kingdom; 9 Saint Vincent College, Latrobe, Pennsylvania, United States of America; 10 Department of Computer Science and Department of Bioengineering, University of Illinois, Urbana-Champaign, Champaign, United States of America; 11 School of Electrical Engineering and Computer Science, Ohio University, Athens, Ohio, United States of America; Princeton University, UNITED STATES

## Abstract

Bioinformatics is recognized as part of the essential knowledge base of numerous career paths in biomedical research and healthcare. However, there is little agreement in the field over what that knowledge entails or how best to provide it. These disagreements are compounded by the wide range of populations in need of bioinformatics training, with divergent prior backgrounds and intended application areas. The Curriculum Task Force of the International Society of Computational Biology (ISCB) Education Committee has sought to provide a framework for training needs and curricula in terms of a set of bioinformatics core competencies that cut across many user personas and training programs. The initial competencies developed based on surveys of employers and training programs have since been refined through a multiyear process of community engagement. This report describes the current status of the competencies and presents a series of use cases illustrating how they are being applied in diverse training contexts. These use cases are intended to demonstrate how others can make use of the competencies and engage in the process of their continuing refinement and application. The report concludes with a consideration of remaining challenges and future plans.

## Author summary

As data size and complexity increase in life science research, so the need for bioinformatics training has increased. This training is required across a wide variety of audiences, but varies in the level of detail and content that needs to be delivered. A scientist wishing to use some bioinformatics tools to analyse their specific dataset will require different competencies than one that provides support in a bioinformatics services environment. The Curriculum Task Force of the International Society of Computational Biology (ISCB) Education Committee has attempted to address this by developing a set of bioinformatics core competencies and mapping these to ten different user profiles across the spectrum of potential trainees. Here we present the final iteration of the competencies and some examples to demonstrate how they have been used to drive bioinformatics curriculum development and training in different settings.

## Introduction

The need for bioinformatics education and training is immense, but it is also diverse. There is a wide range of audiences who are potential recipients of training, each of which has different needs in terms of what skills or knowledge they require and at what depth. For example, someone training to be a bioinformatics engineer (which we define as someone who will actively be involved in the development and application of bioinformatics algorithms) requires in-depth knowledge of existing algorithms, how they work, how to critically evaluate them, and how to interpret the results. By contrast, a bioinformatics user (which we define as someone making use of bioinformatics resources in an applied context, such as in medical practice) would need a basic level of understanding of the methods and a stronger focus on the interpretation of the outputs. In a recent publication [1], the ISCB Education Committee’s Curriculum Task Force described the potential for refinement and application of bioinformatics core competencies for different user groups. Here, we describe the further refinement of these competencies and provide a series of use cases illustrating their applications to different bioinformatics education and training programs globally.

## Development of core competencies for bioinformatics

The ISCB Curriculum Task Force undertook the task of identifying some of the breadth of needs for bioinformatics education, as described in a series of reports from the task force. This effort arose first from a series of surveys of current training practice and desired training needs [2], which identified a set of broad categories of training needs but also widespread disparities across programs in what was taught, how, and for what intended target audiences. An outcome of these surveys was the need for identifying a set of core competencies as broad categories of skills and training that cross different programs and training needs and that can provide a basis for discussing similarities and differences between programs and desired outcomes. This led to a further effort to define a set of initial core competencies [3] that in turn led to an intensive program of community engagement to refine these competencies to better serve the breadth of needs of the bioinformatics training community.

There were three major steps in the development of the core competencies: (1) defining the competencies needed for using bioinformatics, (2) defining a variety of user profiles describing distinct subgroups in need of training, and (3) defining how the competencies will apply to each user profile (scoring). The core competency framework was developed through an iterative process with input from multiple parties from diverse backgrounds with a connection to bioinformatics. In order to gain a broader appreciation of which competencies the bioinformatics community considers relevant for different bioinformatics user profiles, the ISCB Curriculum Task Force has run several competency workshops (discussion sessions for defining the competencies and their applications) both at ISCB conferences and at other bioinformatics education venues such as the GOBLET (Global Organisation for Bioinformatics Learning, Education and Training) Annual General Meeting. Each iteration of a competency workshop has greatly enhanced not only the competencies themselves but also the definitions of the user profiles [1] and the competency-use case scoring mechanism.

Initially, the mapping of bioinformatics competencies to audiences considered three major user profiles: (1) the bioinformatics user; (2) the bioinformatics scientist; and (3) the bioinformatics engineer. Early competency workshops quickly surmised that these user profiles were too narrow and did not adequately capture the breadth of roles requiring bioinformatics competency and curriculum. Participants spent much of the workshop time defining a bioinformatics user or distinguishing a bioinformatics scientist from a bioinformatics engineer. The use case roles were subsequently expanded to better embody the breadth of bioinformatics users, including physicians, lab technicians, ethicists and biocurators, scientists (which include the discovery biologist, academic bioinformatics researcher and core facility scientist), and engineers (which may be a bioinformatician in academia, bioinformatician in research institute, or software engineer). This change allowed for subsequent workshop participants to self-select according to the category of user with which they most identified.

With user profiles better defined, competency workshops then struggled with the competencies themselves and their definitions. Several early competency definitions appeared to overlap. For example, “Apply knowledge of computing appropriate to the discipline (e.g., effectively utilize bioinformatics tools)” closely resembled “Analyze a problem and identify and define the computing requirements appropriate to its solution (e.g., define algorithmic time and space complexities and hardware resources required to solve a problem).” Workshop participants helped to reduce the redundancy in our initial set of bioinformatics competencies from 20 competencies to a refined set of 16 competencies.

Competency workshops have additionally helped to revise the scoring of competencies for each user profile. Early workshops scored the applicability of a bioinformatics competency to a particular profile with a simple yes/no response, which did not allow for an appreciation of the depth of the competency necessary for a given profile. Such a scoring approach, while better than no score, would not be helpful when developing a curriculum for a specific user profile. Subsequent workshops used a graded scoring approach, with grades ranging from 1 (no competency required) to 4 (specialist knowledge required). This, too, proved too ambiguous to allow for meaningful discussion and classification. The scoring approach was thus revised again to the current model, which uses the Bloom’s Revised Taxonomy [4] terms: knowledge, comprehension, application, analysis, synthesis, and evaluation. While the use of Bloom’s Taxonomy has been useful in mapping competency levels to each of the user profiles, this change required refinement of the competency list as several of the earlier competencies incorporated Bloom’s Taxonomy terms.

Overall, competency workshops have been invaluable to the enhancement and refinement of the bioinformatics competencies. Through these workshops, the ISCB Curriculum Task Force has been able to construct a useful set of bioinformatics competencies that curriculum developers can use to develop, compare, and assess impactful bioinformatics training programs for a wide range of audiences and ultimately help establish bioinformatics skills in such audiences [3].

Table 1 reports the current state of the competencies developed and refined through this community engagement process. Tables 2–4 map these refined competencies to a broader set of personas, suggested over the course of the Task Force’s community engagement efforts, via Bloom’s Taxonomy terms. For reference, Table 5 provides examples and definitions of the Bloom's Revised Taxonomy terms. In the next section, we provide some examples of how the competencies have been applied in a variety of training contexts.

**Table 1:** Bioinformatics core competencies. This table provides the current competency list following a process of community engagement. It specifically reflects a significant refinement of the competencies designed to accommodate scoring in terms of Bloom’s Taxonomy.

Label	Competency
A	General biology
B	Depth in at least one area of biology (e.g., evolutionary biology, genetics, molecular biology, biochemistry, anatomy, physiology).
C	Biological data generation technologies.
D	Details of the scientific discovery process and of the role of bioinformatics in it.
E	Statistical research methods in the context of molecular biology, genomics, medical, and population genetics research.
F	Bioinformatics tools and their usage.
G	The ability of a computer-based system, process, algorithm, component, or program to meet desired needs in scientific environments/problem.
H	Computing requirements appropriate to solve a given scientific problem (e.g., system, process, algorithm, component or program; define algorithmic time and space complexities and hardware resources required to solve a problem).
I	GUI/Web-based computing skills appropriate to the discipline (e.g., effectively use bioinformatics and analysis tools through web).
J	Command line and scripting based computing skills appropriate to the discipline.
K	Construction of software systems of varying complexity based on design and development principles.
L	Local and global impact of bioinformatics and genomics on individuals, organizations, and society.
M	Professional, ethical, legal, security, and social issues, and responsibilities of bioinformatics and genomic data in the workplace.
N	Effective communication of bioinformatics and genomics problem/issue/topics with a range of audiences, including, but not limited to, other bioinformatics professionals.
O	Effective teamwork to accomplish a common scientific goal.
P	Engage in continuing professional development in bioinformatics.

**Table 2:** Mapping of competencies to bioinformatics user personas via Bloom’s Taxonomy.

Competency \ Persona	Physician	Lab technician	Ethicist	Biocurator
A. General biology	knowledge to application	comprehension	knowledge	comprehension
B. Depth in at least one area of biology (e.g., evolutionary biology, genetics, molecular biology, biochemistry, anatomy, physiology)	application	application to evaluation	evaluation	application to evaluation
C. Biological data generation technologies.	knowledge	knowledge to evaluation	knowledge	knowledge
D. Details of the scientific discovery process and of the role of bioinformatics in it.	application to analysis	comprehension to analysis	knowledge to comprehension	comprehension to evaluation
E. Statistical research methods in the context of molecular biology, genomics, medical, and population genetics research.	knowledge to application	knowledge to application	knowledge to comprehension	comprehension
F. Bioinformatics tools and their usage.	comprehension	knowledge to analysis	knowledge	application
G. The ability of a computer-based system, process, algorithm, component, or program to meet desired needs in scientific environments/problem.	N/A	knowledge	N/A	comprehension to application
H. Computing requirements appropriate to solve a given scientific problem (e.g., system, process, algorithm, component or program; define algorithmic time and space complexities and hardware resources required to solve a problem).	N/A	knowledge	N/A	comprehension to application
I. GUI/Web-based computing skills appropriate to the discipline (e.g., effectively use bioinformatics and analysis tools through web).	knowledge	application	comprehension	application to evaluation
J. Command line and scripting-based computing skills appropriate to the discipline.	N/A	knowledge	N/A	comprehension
K. Construction of software systems of varying complexity based on design and development principles.	N/A	N/A	N/A	knowledge
L. Local and global impact of bioinformatics and genomics on individuals, organizations, and society.	knowledge	comprehension	application	comprehension
M. Professional, ethical, legal, security and social issues and responsibilities of bioinformatics and genomic data in the workplace.	application	evaluation	evaluation	analysis
N. Effective communication of bioinformatics and genomics problem/issue/topics with a range of audiences, including, but not limited to, other bioinformatics professionals	comprehension	application	application	application to evaluation
O. Effective teamwork to accomplish a common scientific goal.	knowledge	analysis	knowledge	analysis
P. Engage in continuing professional development in bioinformatics.	evaluation to analysis	application	application to evaluation	application

**Table 3:** Mapping of competencies to bioinformatics scientist personas via Bloom’s Taxonomy.

Competency \ Persona	Discovery biologist/ academic life science researcher	Molecular life science educator	Academic bioinformatics researcher	Core facility scientist
A. General biology	evaluation	comprehension	synthesis	knowledge
B. Depth in at least one area of biology (e.g., evolutionary biology, genetics, molecular biology, biochemistry, anatomy, physiology)	evaluation	analysis	evaluation	evaluation
C. Biological data generation technologies.	evaluation	understand	evaluation	evaluation
D. Details of the scientific discovery process and of the role of bioinformatics in it.	application	evaluation	synthesis to evaluation	application
E. Statistical research methods in the context of molecular biology, genomics, medical, and population genetics research.	application	evaluation	synthesis to evaluation	application
F. Bioinformatics tools and their usage.	application	evaluation	synthesis to evaluation	application
G. The ability of a computer-based system, process, algorithm, component, or program to meet desired needs in scientific environments/problem.	application	comprehension	synthesis to evaluation	evaluation
H. Computing requirements appropriate to solve a given scientific problem (e.g. system, process, algorithm, component or program; define algorithmic time and space complexities and hardware resources required to solve a problem).	application	comprehension	synthesis to evaluation	evaluation
I. GUI/Web-based computing skills appropriate to the discipline (e.g., effectively use bioinformatics and analysis tools through web).	application	comprehension	comprehension	evaluation
J. Command line and scripting-based computing skills appropriate to the discipline.	application	comprehension	application	evaluation
K. Construction of software systems of varying complexity based on design and development principles.	comprehension	comprehension	synthesis to evaluation	application
L. Local and global impact of bioinformatics and genomics on individuals, organizations, and society.	knowledge	comprehension	comprehension	remember
M. Professional, ethical, legal, security and social issues and responsibilities of bioinformatics and genomic data in the workplace.	application	comprehension	application	application
N. Effective communication of bioinformatics and genomics problem/issue/topics with a range of audiences, including, but not limited to, other bioinformatics professionals	application	comprehension	synthesis to evaluation	application
O. Effective teamwork to accomplish a common scientific goal.	application	analysis	evaluation	application
P. Engage in continuing professional development in bioinformatics.	application	application	application	application

**Table 4:** Mapping of competencies to bioinformatics engineer personas via Bloom’s Taxonomy.

Competency \ Persona	Bioinformatician in an academic or research infrastructure support role	Bioinformatics software developer/ software engineer
A. General biology	application	application
B. Depth in at least one area of biology (e.g., evolutionary biology, genetics, molecular biology, biochemistry, anatomy, physiology)	comprehension	comprehension
C. Biological data generation technologies.	comprehension	comprehension
D. Details of the scientific discovery process and of the role of bioinformatics in it.	application	application
E. Statistical research methods in the context of molecular biology, genomics, medical, and population genetics research.	application	application to synthesis
F. Bioinformatics tools and their usage.	evaluation	evaluation
G. The ability of a computer-based system, process, algorithm, component, or program to meet desired needs in scientific environments/problem.	evaluation	evaluation
H. Computing requirements appropriate to solve a given scientific problem (e.g., system, process, algorithm, component or program; define algorithmic time and space complexities and hardware resources required to solve a problem).	synthesis	synthesis to evaluation
I. GUI/Web-based computing skills appropriate to the discipline (e.g., effectively use bioinformatics and analysis tools through web).	evaluation	evaluation
J. Command line and scripting-based computing skills appropriate to the discipline.	analysis	analysis to evaluation
K. Construction of software systems of varying complexity based on design and development principles.	analysis	analysis to evaluation
L. Local and global impact of bioinformatics and genomics on individuals, organizations, and society.	comprehension	comprehension
M. Professional, ethical, legal, security and social issues and responsibilities of bioinformatics and genomic data in the workplace.	comprehension	comprehension
N. Effective communication of bioinformatics and genomics problem/issue/topics with a range of audiences, including, but not limited to, other bioinformatics professionals	application	application
O. Effective teamwork to accomplish a common scientific goal.	application	application to analysis
P. Engage in continuing professional development in bioinformatics.	application	application to analysis

**Table 5:** Bloom’s revised Taxonomy. The table provides, for each term, illustrative examples of skills demonstrating the given level of competency and a general definition.

Cognitive Level	Illustrative Verbs	Definitions
Knowledge	arrange, define, describe, duplicate, identify, label, list, match, memorize, name, order, outline, recognize, relate, recall, repeat, reproduce, select, state	remembering previously learned information
Comprehension	classify, convert, defend, discuss, distinguish, estimate, explain, express, extend, generalize, give example(s), identify, indicate, infer, locate, paraphrase, predict, recognize, rewrite, report, restate, review, select, summarize, translate	grasping the meaning of information
Application	apply, change, choose, compute, demonstrate, discover, dramatize, employ, illustrate, interpret, manipulate, modify, operate, practice, predict, prepare, produce, relate schedule, show, sketch, solve, use write	applying knowledge to actual situations
Analysis	analyze, appraise, breakdown, calculate, categorize, classify, compare, contrast, criticize, derive, diagram, differentiate, discriminate, distinguish, examine, experiment, identify, illustrate, infer, interpret, model, outline, point out, question, relate, select, separate, subdivide, test	breaking down objects or ideas into simpler parts and seeing how the parts relate and are organized
Synthesis	arrange, assemble, categorize, collect, combine, comply, compose, construct, create, design, develop, devise, explain, formulate, generate, plan, prepare, propose, rearrange, reconstruct, relate, reorganize, revise, rewrite, set up, summarize, synthesize, tell, write	rearranging component ideas into a new whole
Evaluation	appraise, argue, assess, attach, choose, compare, conclude, contrast, defend, describe, discriminate, estimate, evaluate, explain, judge, justify, interpret, relate, predict, rate, select, summarize, support, value	making judgments based on internal evidence or external criteria

### Use cases

To better illustrate the use of the competencies, we present here a series of brief use cases—scenarios in which the competencies have proven valuable already in defining, refining, or assessing a bioinformatics training mechanism. These use cases were selected to highlight a diverse set of training needs, user personas, types of training programs, and educational settings. In this spirit, we present examples grouped into three categories: (1) complete degree programs for which the competencies have proven valuable to overall curriculum design or refinement; (2) supplements to existing degree programs (i.e., specializations, tracks, certificates); and (3) training resources outside the context of specific degree programs.

### Degree programs

#### Introductory and masters bioinformatics training in Africa: H3ABioNet

H3ABioNet (www.h3abionet.org), a Pan African bioinformatics network for H3Africa [5], has developed a bioinformatics training program for African scientists from the Human Heredity and Health in Africa (www.h3africa.org) consortium. This involves bioinformatics training for a broad range of audiences, primarily in genomics data analysis, and the development of new bioinformatics degrees to train bioinformatics scientists. Though there are some institutions in Africa offering bioinformatics postgraduate degrees, this was limited to a handful of countries, and many additional institutions expressed a desire to develop and offer such degrees in order to build the next generation of bioinformatics academics. An African Bioinformatics Education Committee was established along with a Curriculum Task Force, which set about designing a bioinformatics master’s program. Topic areas were selected from existing master’s courses and those proposed in [3]. From these, core modules were defined and augmented with additional elective modules relevant to specific institutions, based on their research priorities. The Curriculum Task Force then fleshed out the detailed content of each module, and started mapping these to core competencies required of a bioinformatics specialist. Though the focus of some master’s programs may vary from the more biological to a stronger emphasis on software engineering, there were common competencies with which all bioinformatics master’s graduates should be equipped. While some African institutions have specific research focus areas, the feeling was that all students training to be bioinformaticians should be exposed to a set of core subjects, which are in line with the ISCB’s recommendations, and the elective subjects then tend to be dependent on the research focus. The proposed curriculum has been put into practice, with at least two universities in Africa starting their first master’s programs in the last 2 years.

For bioinformatics users, H3ABioNet has successfully run several specialist short courses to train researchers on next generation sequence analysis, metagenomics, genome wide association studies, and other topics. However, through interactions with users, there emerged a need for more basic “introduction to bioinformatics” training. In response, H3ABioNet developed an Introduction to Bioinformatics course delivered remotely to classrooms across multiple countries. The curriculum was derived primarily from topics used for the master’s courses, but this time mapping it to competencies for bioinformatics users and removing topics with a modelling or programming focus. The competencies for this audience are thus more focussed on a basic understanding of the topic, example algorithms, and how the tools can be applied to answer biological questions. The practicals are also designed to enable users to navigate their way through the tools and learn to interpret the outputs. This course was run successfully for the first time in 2016 and was assessed to determine whether the required core competencies were acquired.

Using core competencies for both cases described above enabled course organisers to better define the detailed content, contact hours, and focus for each module, based on the intended audience. We could also use the competencies to define learning outcomes and refine module assessments.

#### Undergraduate and graduate degree programs in a US research university: Computational biology education at Carnegie Mellon

Carnegie Mellon University has long been active in education in computational biology and bioinformatics, providing several opportunities for considering how a general set of competencies can apply to diverse populations. These experiences include degree programs in computational biology at several levels, including a BS in computational biology (since 1989), an MS in computational biology (since 1999), a PhD in computational biology (offered jointly with the University of Pittsburgh since 2005), and required training in computational biology as part of the core of the BS in biological sciences, the university's general undergraduate biology major. While all of these programs predate the ISCB competencies, the competencies provide a basis for considering how well these programs prepare students for work involving computational biology to differing degrees. Two of these programs—the BS in biological sciences and the PhD in computational biology—are discussed as examples of programs with very different student populations and training needs that can be evaluated in light of the competencies.

Carnegie Mellon's BS in biological sciences illustrates one kind of bioinformatics training: for students primarily training for work in experimental biology. Carnegie Mellon took the still unusual step in 2013 of making Introduction to Computational Biology (ICB) a core requirement of every undergraduate biological sciences major, providing an opportunity to explore how one would design a class to be accessible but rigorous and useful to a population of general biology students. Applying these competencies, then, requires working in the context of students who are typically taking a single class on computational biology but within a full undergraduate biology curriculum. Some competencies, primarily those focused on technical aspects of computational biology, can be covered reasonably well at the level needed by an experimental biologist within a single computational biology class (C,D,F,I,J; see Table 1). Other important areas, such as more conventional biological knowledge, are covered thoroughly in other areas of an undergraduate biological sciences curriculum, e.g., in more traditional core classes such as Genetics, Biochemistry, or Cell Biology (A,B). Still others, such as the topics that fall broadly under communications and professional development, are covered elsewhere in the curriculum by a variety of mechanisms inside and outside the classroom (M,N,O,P). Still other areas go beyond what can fit in one introductory class but are also not covered elsewhere. Some of these (G,H,K) are competencies that may not be needed by this population but can be flagged for consideration in revisions of ICB. The most interesting topics are those that are crucial for experimental biologists, cannot be covered sufficiently in ICB, and are not covered elsewhere (E, i.e., biostatistics). ICB gives this latter area enough coverage to convey the key ideas needed for bioinformatics work, but the competencies flag it as an area in need of further development in the curriculum as a whole.

The Carnegie Mellon/University of Pittsburgh joint PhD in computational biology offers an example at another extreme of the spectrum: a full multi-year training program for students expected to become experts in computational biology, who are expected to graduate competent to lead independent research programs in the area, teach computational biology, run bioinformatics core facilities, or pursue similarly demanding jobs. Computational biology programs face a special challenge compared with more traditional degree programs, in that the lack of clear standards for training at the undergraduate level means that there is little one can assume or enforce about background knowledge of incoming students beyond basic competencies in biology, computing, and mathematics. Furthermore, since a PhD program is research-focused and under pressure to limit time to degree, formal training can occupy only a finite amount of a student's time, equivalent to roughly a year of full-time coursework. To a limited degree, the program can rely on admissions standards, remediation, and self-teaching to assume some basics of all students (A,F,I,J). Some competencies can be handled by flexible menu-based requirements to meet a competency in ways appropriate to each student’s individual needs and background (B). In others, every student needs a high level of competency and this must be met with specialized core classes designed for this population (C,D,E,G,H). Others must be met within the curriculum through specialized professional development mechanisms as well as one-on-one mentorship by the thesis advisor (K,L,M,N,O,P). Nonetheless, some competencies, especially those that depend on the mentorship of the research advisor, may be acquired much more effectively by some students than others. The competencies again suggest that these topics should be flagged for consideration for more formal training in the future. Furthermore, the challenges faced by this program with respect to knowledge of incoming students make clear the value that accepted standards for competencies at the undergraduate level could have in making most effective use of time in graduate school for specialists in the field.

#### Undergraduate training in an Australian university: Bioinformatics engineering education at the University of New South Wales (UNSW)

The University of New South Wales (UNSW) (Sydney, Australia) offers a Bachelor of Engineering (Bioinformatics Engineering) program, which aims to empower graduates to design and implement computing systems for bioinformatics, including software algorithms as well as data management and analysis infrastructures. The BE (Bioinformatics Engineering) degree started in 2001 and is the longest-running undergraduate bioinformatics program in Australia. It is fully accredited as an engineering degree by Engineers Australia: graduates are recognized as entry-level engineers in all the countries that are signatories of the Washington Accord—an international agreement among bodies responsible for accrediting engineering degree programs [6]. The program is revised periodically to keep it relevant and is reviewed every 5 years by an external panel of engineers to ensure that accreditation criteria are met. Curriculum mapping of the program content to the ISCB and Engineers Australia core competencies as well as to the university’s Graduate Attributes is a crucial step in that process.

The process starts at the whole program level, by identifying which courses in the program significantly address specific core competencies. Then, for each core competency, the learning outcomes of the relevant courses are examined and refined to address this competency. Assessment activities are tailored with the core competency in mind to ensure that at the conclusion of the course, students are able to demonstrate that they have achieved sufficient levels of proficiency. The process is repeated for each core competency, resulting in a matrix mapping competencies to curricula. The matrix may reveal weaknesses, which can be addressed by modifying or substituting courses. For example, in the most recent revision, the program was modified to replace generic elective courses with additional design project courses and software engineering workshops. To facilitate the evaluation of a program relative to core competencies and graduate attributes, the university’s Academic Information Management System requires each course description to include a mapping of the course’s learning outcomes to both assessment tasks and core competencies. The competency mapping matrix can then be generated automatically for each course and at a whole program level. Expanding the ISCB curriculum guidelines by including examples of learning outcomes for each core competency would facilitate this kind of analysis and increase the usefulness of the competencies in curriculum design and evaluation.

In addition to its long-standing Bachelor of Engineering in bioinformatics, UNSW recently introduced a Bachelor of Science Bioinformatics major emphasising the use of existing bioinformatics methods for biological discovery rather than the design of new bioinformatics methods. The core competencies were used to guide the design of the program by identifying the competencies to emphasize relative to the Engineering program (B, C, and D) and those for which a lower level of achievement was acceptable (G, H, J, K, M, O). This in turn guided the choice of courses for the Bachelor of Science major.

#### Undergraduate degrees in bioinformatics at a small liberal arts college: Saint Vincent college

The bioinformatics program at Saint Vincent College, a small liberal arts college in western Pennsylvania, was started in 2005. The program is small, with less than 20 students in the major, but it has graduated at least one student each year from 2009 to the present. Initially, there was only one set of required courses for the BS degree, which included courses covering programming (in C++), data structures, discrete structures, introduction to databases, biostatistics, cell biology, molecular genetics, genomics, and biomedical informatics. There was also a capstone three-semester research project. Roughly speaking, three types of students entered the program: (1) students who enjoyed both biology and computation and were good at both; (2) students who enjoyed biology but struggled with the programming courses; and (3) students who enjoyed programming but struggled in the upper biology courses, particularly labs. The program tended to lose students in the latter two groups from the program to biology or computer science. As a result, in 2013 they split the curriculum into two tracks—biology and computation—to try to accommodate students in these groups and keep them in the major. About two-thirds of the courses are common between the two tracks, but, for example, the biology track only requires one semester of C++ programming rather than three for the computation track.

In 2015, the program underwent a comprehensive program review, including both internal reviewers and an external reviewer. As part of the initial report on the program, the ISCB Core Competencies were used as a standard against which to evaluate the curriculum and student training, which was very valuable as without them, it would have been difficult to find a way to evaluate strengths and weaknesses of the curriculum against an external standard. One of the issues raised in the review was the learning goals for the major and how those relate to the two tracks, since the learning goals had not been revised when the two tracks were implemented. Roughly speaking, the two tracks correspond with the ISCB roles of bioinformatics users and bioinformatics scientists. These issues, examined in light of the competencies, highlight a principal challenge for smaller programs: how to accommodate both types of students given limitations on number of faculty, types of courses available from different departments, enrollment, etc.

### Certifications, tracks, and specializations

#### Certificate programs and specializations: Ohio university

Ohio University offers bioinformatics certificates at both the undergraduate and graduate levels. Additionally, computer science students at the BS, MS, and PhD degree levels may specialize in bioinformatics by selecting degree tracks that contain appropriate biology and bioinformatics courses. To complete an undergraduate bioinformatics certificate, trainees take courses in the following: statistics, discrete mathematics, data structures, genetics, laboratory biology, cell biology, one elective course in biology, bioinformatics tools, and data mining. A graduate certificate in bioinformatics is earned by completing graduate level courses in biochemistry, two elective courses in genetics/molecular biology/systematics, laboratory biology, bioinformatics tools, computational genomics, data mining, or statistical foundations for bioinformatics. Similarly, explicit biomedical informatics tracks within the computer science degree programs allow students to elect a structured training program.

The elucidation of the training categories of bioinformatics engineer, scientist, and user necessitates a review of Ohio’s programs. While the bioinformatics specializations within the computer science degree programs provide adequate training for bioinformatics engineers, it would be beneficial to migrate from one-size-fits-all bioinformatics certificate programs to multi-track programs for training bioinformatics users, scientists, and engineers. The certificate programs are currently being broadened to allow customization for training in each different bioinformatics role. As an initial step, the biology elective course requirement is being changed to a role-specific elective course requirement. This will allow bioinformatics engineers to select elective courses in algorithm analysis, data science, database design, machine learning, artificial intelligence, software engineering, computer security, and parallel computing. Additionally, the bioinformatics certificate program requirements are being redesigned to feature specific tracks for users, scientists, and engineers. This redesign process would be aided by having the ISCB competencies for bioinformatics engineers detailed, perhaps in the form of sample programs (e.g., an aggregation from the survey of bioinformatics programs discussed in [1]), or by mapping each competency to suggested courses and/or course topics (e.g., from the controlled vocabulary defined in [1]).

#### Specialist track in an undergraduate bioengineering program: The University of Illinois

At the University of Illinois, undergraduate bioengineering majors select a track, one of which is Computational and Systems Biology (CSB). Students not in the CSB track get a small amount of programming experience, but do take a non-majors CS course in their sophomore year that exposes them to MATLAB and C programming. They also take a junior-level course, Computational Tools for Biological Data, that covers basic probability and statistics; hypothesis testing; modelling and simulation; and experimental design and applies these concepts and techniques to human genomic variation; sequence alignment; Hidden Markov Models and gene finding; cancer genomics; and gene regulatory networks. Students in the CSB track take the Computational Tools for Biological Data course described above but have a more rigorous training in mathematics and computer science. Specifically, CSB students take courses for CS majors, including introductory programming, discrete mathematics, data structures, data mining and bioinformatics. Overall, CSB students have a rigorous training in mathematics, probability, statistics, and computer science, and take at least two senior-level courses in which techniques from these disciplines are applied in bioinformatics analyses. Experience with this population highlights a gap remaining in the competencies, with a population not currently well represented in their use. It suggests a possible direction for future work, as the Bioinformatics Engineer Curriculum Working Group might extend its guidelines to better encompass the field of bioengineering.

### Other training guidelines and resources

#### Bioinformatics short courses: European Bioinformatics Institute (EMBL-EBI) and university of Cambridge

Both EMBL-EBI (www.ebi.ac.uk/training) and the University of Cambridge (UCAM, http://bioinfotraining.bio.cam.ac.uk/) offer extensive programmes of short courses that enable the research community to gain competency in bioinformatics. These programmes differ from the full-time curricula described above in that they are aimed at individuals already pursuing a research career. Most of the scientists attending these courses are PhD students, postdoctoral researchers, or more senior researchers (in academia or in industry), who are performing data-intensive experiments and need guidance on experimental design, data analysis, and interpretation. As a proof of principle for ELIXIR, Europe’s distributed infrastructure for biological data with nodes in 20 countries, EMBL-EBI and UCAM recently performed an exercise to map their course programmes to the ISCB competency framework. The goal was to identify any gaps in training provision and also to rapidly check the robustness of the competency profile—in total they looked at 50 short courses offered by UCAM and 21 at EMBL-EBI, covering a wide range of topics aimed primarily at bioinformatics scientists and bioinformatics users. Both programmes included coverage of all the competency areas, with only a very small number of courses increasing competence in A, general biology (this is already well developed in the target audience, many of whom have postgraduate degrees in the biological sciences) and a high proportion of the courses increasing competence in F, bioinformatics tools and their usage (48 courses from UCAM; 20 courses from EMBL-EBI); D, details of the scientific discovery process and the role of bioinformatics in it (34 courses from UCAM; 20 courses from EMBL-EBI); and N, effective communication of bioinformatics problems, issues and topics (28 courses from UCAM; 20 from EMBL-EBI). Two competency areas were identified that they felt were not adequately covered by the existing framework and that they would like to propose adding: Data curation for dissemination of research data (for example, the annotation of data required when submitting data sets to public databases, and the annotation of data performed by professional biocurators who add value to these resources) and data curation for analysis of research data (for example, annotation of a newly sequenced genome to find orthologues/paralogues or to gain a functional overview of the genome). This exercise, if performed across all of the ELIXIR nodes, will help to understand the impact of ELIXIR’s training portfolio for different target audiences and will enable them to shape our offering accordingly. Mapping existing short courses to bioinformatics core competencies could also be used to help individuals along a learning path, taking them from one competency level to the next.

#### Clinical bioinformatics: The United Kingdom 100,000 genomes project

The need for bioinformatics to infiltrate current clinical practice is urgent, expedited by programs such as the 100,000 Genomes Project in the UK (https://www.genomicsengland.co.uk/the-100000-genomes-project/), which will sequence 100,000 patient genomes with the goal of using the genomic data to inform clinical decision-making. Many different types of healthcare professionals will be impacted by this project. For example, specialist healthcare scientists require training to handle and interpret genomic data; clinical staff involved in recruiting patients to the 100,000 Genomes Project require training to understand the results of genome sequencing and to counsel patients (and their relatives); and the general workforce requires training to provide awareness of genomic medicine and how it can improve patient care. To this aim, in 2014 Health Education England convened a “Task and Finish Group” in clinical bioinformatics to provide recommendations on training requirements arising not only as an immediate consequence of the 100,000 Genomes Project but also from the increasing use of biomolecular data in medical practice as a whole. The group decided to tackle the immediate problem by defining the competencies needed by healthcare professionals to enable them to use data emerging from the 100,000 Genomes Project to inform clinical decision-making. As a proof of principle, the group also mapped these competencies to existing or newly designed training programmes commissioned by Health Education England, to inform the design of future training programmes for healthcare professionals.

As a starting point, the group used the ISCB core competencies and a policy paper that defined the role of clinical bioinformaticians to draft a rough list of competencies; the group also created a list of different types of healthcare professionals likely to be impacted by the 100,000 Genomes Project. Each member of the group then consulted with colleagues and the wider community, asking them to provide information on which competencies were required to make use of the 100,000 genomes data, and requesting participants to think about whether any additional competencies are required. At least five representatives of each profession were consulted, and all input was combined to create a consensus competency profile. This consensus view, published in a white paper, ‘Developing clinical bioinformatics training in the NHS’ (https://www.genomicseducation.hee.nhs.uk/images/publications/Developing_NHS_Clinical_Bioinformatics_Training.pdf), captures not only which competencies are required by the professions listed but also an indication of the level of expertise required, from no knowledge through general awareness and working knowledge to specialist expertise. The profile does not provide guidance on the evidence required to assess whether an individual has gained each of the required competencies, but this would be an obvious next step.

#### Learning framework for a metacurricular resource: The CourseSource bioinformatics learning framework

*CourseSource* (http://www.coursesource.org) is “an open-access journal of peer-reviewed teaching resources for undergraduate biological sciences” [7]. *CourseSource* organizes its resources by biological disciplines (e.g., evolution, genetics, molecular biology, bioinformatics) that play integral roles in biology. Each discipline has an associated framework of learning goals and objectives that undergraduate students in the biological sciences should have reached by the time they have completed their degree. The ISCB curriculum and competency guidelines were used as a model to develop the Bioinformatics Learning Framework. The framework can be viewed at http://www.coursesource.org/courses/bioinformatics. It represents a practical application of the guidelines and provides an elaboration of the guidelines to a level appropriate for implementation in classroom settings.

## Discussion and conclusions

The work of the Task Force identified a pressing need for bioinformatics education but also tremendous variability in the details of this need and widespread confusion about how to meet it for diverse target user populations and training contexts. The effort to develop and successively refine a set of core competencies for bioinformatics training has sought to assist educators in this domain by providing a conceptual framework in which the field can more productively share experiences and pool our efforts in identifying best practices for bioinformatics education in the face of divergent needs and expectations. Several years of community engagement efforts and subsequent refinements have brought us ever closer to that goal, leading to a broader appreciation of the range of user personas in need of bioinformatics education and a more productive language through which to identify and discuss shared needs and training mechanisms. As the use cases presented here illustrate, the core competencies that arose from this process provide a basis for the community of bioinformatics educators, despite widely divergent goals and student populations, to draw upon their common experiences in designing, refining, and evaluating their own training programs.

We caution that these core competencies are not, and are not intended to be, a prescription for a specific set of curricula or curricular standards. While the competencies highlight common points of focus across training scenarios, few points escape dissent. The field is still figuring out what it means to be trained in bioinformatics or how best to provide that training. We do not expect that state of affairs to end in the near future. Nonetheless, we hope that having a framework in which we can evaluate how different programs define and service their training needs will prove valuable in the maturation of bioinformatics as a discipline.

In the future, the Task Force plans to detail its guidelines in a manner similar to the CourseSource framework. Specifically, the plan is to provide an explicit mapping between the competencies and the CourseSource framework, which is tailored for life scientists. The taskforce’s ultimate goal is to have explicit mappings of courses to competencies for each of the personas in the ISCB competency framework. This is already underway for life scientists (with the CourseSource framework) and clinical practitioners (with the NHS clinical bioinformatics framework). Where there are synergies with other frameworks, we see potential to map these to curricula for other personas; for example, the Edison framework for data science has many elements relevant to bioinformatics engineers; the ABET framework was indeed used as a basis to develop the ISCB competency framework; and the curricula described in this manuscript also provide specific examples that can be generalised into a framework for bioinformatics engineers.
